# Sexual and non-sexual social preferences in male and female white-eyed bulbuls

**DOI:** 10.1038/s41598-017-06239-3

**Published:** 2017-07-19

**Authors:** Bekir Kabasakal, Miroslav Poláček, Aziz Aslan, Herbert Hoi, Ali Erdoğan, Matteo Griggio

**Affiliations:** 1 Akdeniz University, Science Faculty, Department of Biology, Antalya, Turkey; 2 Konrad Lorenz Institute of Ethology, Department of Integrative Biology and Evolution, University of Veterinary Medicine, Vienna, Austria; 3 Akdeniz University, Education Faculty, Department of Primary Education, Antalya, Turkey; 4 University of Padova, Department of Biology, Padova, Italy

## Abstract

While the function of ornaments shaped by sexual selection is to attract mates or drive off rivals, these signals may also evolve through social selection, in which the social context affects the fitness of signallers and receivers. Classical ‘mate choice’ experiments often reveal preferences for ornaments, but few studies have considered whether these are strictly sexual or reflect general social preferences. Indeed, an alternative possibility is that ornaments evolve through ‘non-sexual social selection’ (hereafter ‘social selection’). We examined the role of ornamentation (yellow ventral patch) and familiarity (individuals recognize group mates with which they have had previous interactions) on mate choice (opposite-sex stimuli preference) and social choice (same-sex stimuli preference) in both male and female white-eyed bulbuls (*Pycnonotus xanthopygos*). In the mate choice test, females preferred unfamiliar males with increased yellow. There were no biologically important differences in male preferences based on familiarity or intensity of patch colour. In the social choice test, females preferred to associate with familiar females. Males preferred to associate with familiar males but also preferred to associate with less ornamented males. Our results suggest that ornamentation and familiarity are important features, playing different roles in males and females, in both social and sexual selection processes.

## Introduction

Mate choice is suggested to be one driving force of sexual selection responsible for the evolution of exaggerated ornaments^1^. However, many animals also use ornaments, particularly colour traits, to communicate outside a sexual context. Therefore, if particular ornaments confer social advantages, such as competition for non-reproductive resources, social status or prestige^2, 3^, then they may evolve outside the framework of sexual selection. This alternative possibility, a form of ‘non-sexual social selection’ (hereafter ‘social selection’)^4^, has received surprisingly little attention^5–8^, yet may provide adaptive explanations for ornaments in mutually ornamented species if ornamentation confers social benefits^9, 10^. In principle, one can imagine that traits evolved originally throughout social selection can also acquire importance in mate choice. On the other hand, traits evolved initially in the context of sexual selection can also play a role in nonsexual interactions. Alternatively, it could be that sexual and social preferences use different traits or follow different rules. Despite the intuitive importance of the distinction between sexual and social preferences in gregarious species, very few studies have investigated these alternatives^11, 12^. In this context, social familiarity (individuals recognize group mates with which they have had previous interactions) seems to be a relevant factor for investigating how traits may be evaluated in both sexual and social contexts. Indeed, familiarity has been shown to be important in both contexts. Recognizing familiar conspecifics based on visual^13–15^, acoustic^16, 17^ and odour characteristics^18, 19^ is likely to be beneficial in the context of shared antipredator behaviour^20–23^, exploration^24^ or the optimization of foraging efficiency^25–27^. The ability to discriminate familiar and unfamiliar conspecifics may be particularly well developed in social species. At the same time, familiarity seems to affect mate choice in many animals as well, although the results are controversial^28^. More specifically, mating patterns that favour unfamiliar mates have been documented in several studies^29–31^, with such a preference being usually explained in terms of reducing inbreeding while also increasing genetic diversity in offspring^32^. However, some studies have revealed that females are more likely to mate with familiar males^33–36^, and that mating with familiar individuals of the opposite sex seems to have a positive effect on breeding success^37^. Recently, Senar *et al*.^36^ discovered that familiarity interacts with ornament preferences. Their results suggest that both familiarity and sexual ornaments are important in female mate choice. Mate choice for familiar individuals may have important evolutionary implications because it can favour local adaptation and speciation.

In this study, therefore, we examine the role and interaction of ornamentation and familiarity in mate choice and social choice in both male and female white-eyed bulbuls (*Pycnonotus xanthopygos*). The white-eyed bulbul is originally described from the Lebanon and found from the Arabian Peninsula to the Turkish Mediterranean region, inhabiting gardens, palm groves, fruit gardens, scrubs, and open or mixed forest, usually at low altitudes (up to 400 m a.s.l. with the highest known breeding localities at 750 m a.s.l). They breed very densely along the coastal band of the Mediterranean Sea^38, 39^. The northernmost distribution of the species occurs in the Mediterranean region of Turkey. White-eyed bulbul is invasive to Turkey and its range is expected to expand further into northern Anatolia^40, 41^.

The territorial and socially monogamous white-eyed bulbul is an ideal model species for this study. Pair bonds in this species persist throughout the breeding season, over the whole year or frequently through multiple years, which makes the choice of a reproductive partner important. On the other hand, particularly outside the reproductive season, bulbuls flock in social units of about 5 to 50 individuals, suggesting that their social environment is important as well^42^. Furthermore, both sexes are similarly ornamented, with the yellow ventral side (see Fig. S1) is a key trait for mate choice. Indeed, in this species there is assortative mating based on yellow ventral colour and individuals (both males and females) with more yellow in their ventral patches start to breed earlier than duller individuals (own unpublished results). We measured both male and female preferences for familiarity and/or experimentally manipulated ornaments in an aviary four-choice test. To understand the ornament’s importance in sexual or social contexts, we determined i) the relative importance of the ornament, namely intensity of the yellow ventral plumage feathers and familiarity, and ii) whether these preferences are expressed only in opposite-sex (sexual context) or also in same-sex associations (social context). To our knowledge, this is the first attempt to investigate how familiarity and sexual ornamentation interact to affect choice in both sexual and non-sexual social contexts.

## Results

### Experimental manipulations

There were no significant differences between the four stimulus groups (familiar with increased yellow, familiar with decreased yellow, unfamiliar with increased yellow and unfamiliar with decreased yellow) in body mass, tarsus and wing length of males in female mate choice (ANOVA, all *P* > 0.59, all *ω* ^*2*^ < −0.03, n = 36) and female social choice (ANOVA, all *P* > 0.16, all *ω* ^*2*^ < 0.06, n = 40) or females in male mate choice (ANOVA, all *P* > 0.24, all *ω* ^*2*^ < 0.04, n = 31) and male social choice (ANOVA, all *P* > 0.19, all *ω* ^*2*^ < 0.06, n = 30). Thus body size differences between stimulus birds are unlikely to affect choice of focal bird in our experiment.

The treatments, increased yellow (artificially enhanced) stimuli were treated with yellow and the decreased yellow (artificially dulled) stimuli with white non-toxic hair colours applied on the yellow ventral patch, effectively changed the plumage reflectance (difference between before and after manipulation). The yellow chroma was significantly increased by manipulation with yellow hair colour (paired t-test, *t*_*14*_ = −10.52, *P* < 0.001) while white hair colour significantly decreased yellow chroma (paired t-test, *t*_*14*_ = 6.025, *P* < 0.001). Both treatments with hair colours significantly reduced the UV-chroma (increased yellow: paired t-test, *t*_*14*_ = 14.10, *P* < 0.001; decreased yellow: paired t-test, *t*_*14*_ = 2.17, *P* = 0.04). Finally, only white hair colour affected the total brightness (increased yellow: paired t-test, *t*_*14*_ = 0.45, *P* = 0.65; decreased yellow: paired t-test, *t*_*14*_ = −11.07, *P* < 0.001).

### Mate choice

**Figure 1:**
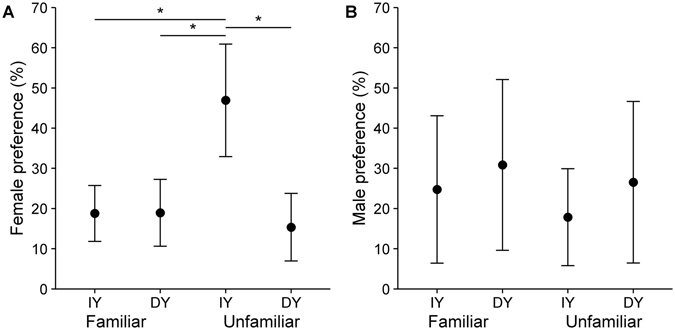
Results of mate choice experiments on white-eyed bulbuls (*Pycnonotus xanthopygos*) using four stimuli with increased or decreased yellow ventral plumage colour (IY: Increased yellow, ID: Decreased yellow) and familiarity. Means presented as dots with 95% CL. Significant differences are marked with an asterisk (Tukey’s post hoc test). (**A**) female mate choice (**B**) male mate choice.

Males and females were equally motivated with no differences between sexes in the time they spent in the choice area (Welch’s t-test: *t*_*29.16*_ = 0.64, *P* = 0.52). In the mate choice test, females significantly preferred unfamiliar males with increased yellow (Fig. 1A, Table 1 and Table S1). However, there were no biologically important differences in male preferences based on familiarity or intensity of patch colour (Fig. 1B, Table 1).

**Table 1:** Results of ANOVA comparing preference of experimental birds, white-eyed bulbuls (*Pycnonotus xanthopygos*), for familiarity and ornament colouration (increased or decreased yellow ventral plumage colour) in sexual and social context, using four different stimuli.

Experiment	Factor	df	f	p	ω²
**Mate choice**					
Females choosing males	Familiarity	1,68	5.89	0.01	0.05
	Yellow	1,68	11.29	0.01	0.10
	Familiarity x Yellow	1,68	10.41	0.01	0.09
Males choosing females	Familiarity	1,56	0.28	0.6	−0.01
	Yellow	1,56	1.04	0.31	0.01
	Familiarity x Yellow	1,56	0.01	0.94	−0.01
**Social choice**					
Males choosing males	Familiarity	1,44	6.04	0.01	0.08
	Yellow	1,44	4.08	0.05	0.05
	Familiarity x Yellow	1,44	2.97	0.09	0.03
Females choosing females	Familiarity	1,72	7.95	0.01	0.08
	Yellow	1,72	0.96	0.33	−0.01
	Familiarity x Yellow	1,72	0.79	0.37	−0.01

### Social choice

**Figure 2:**
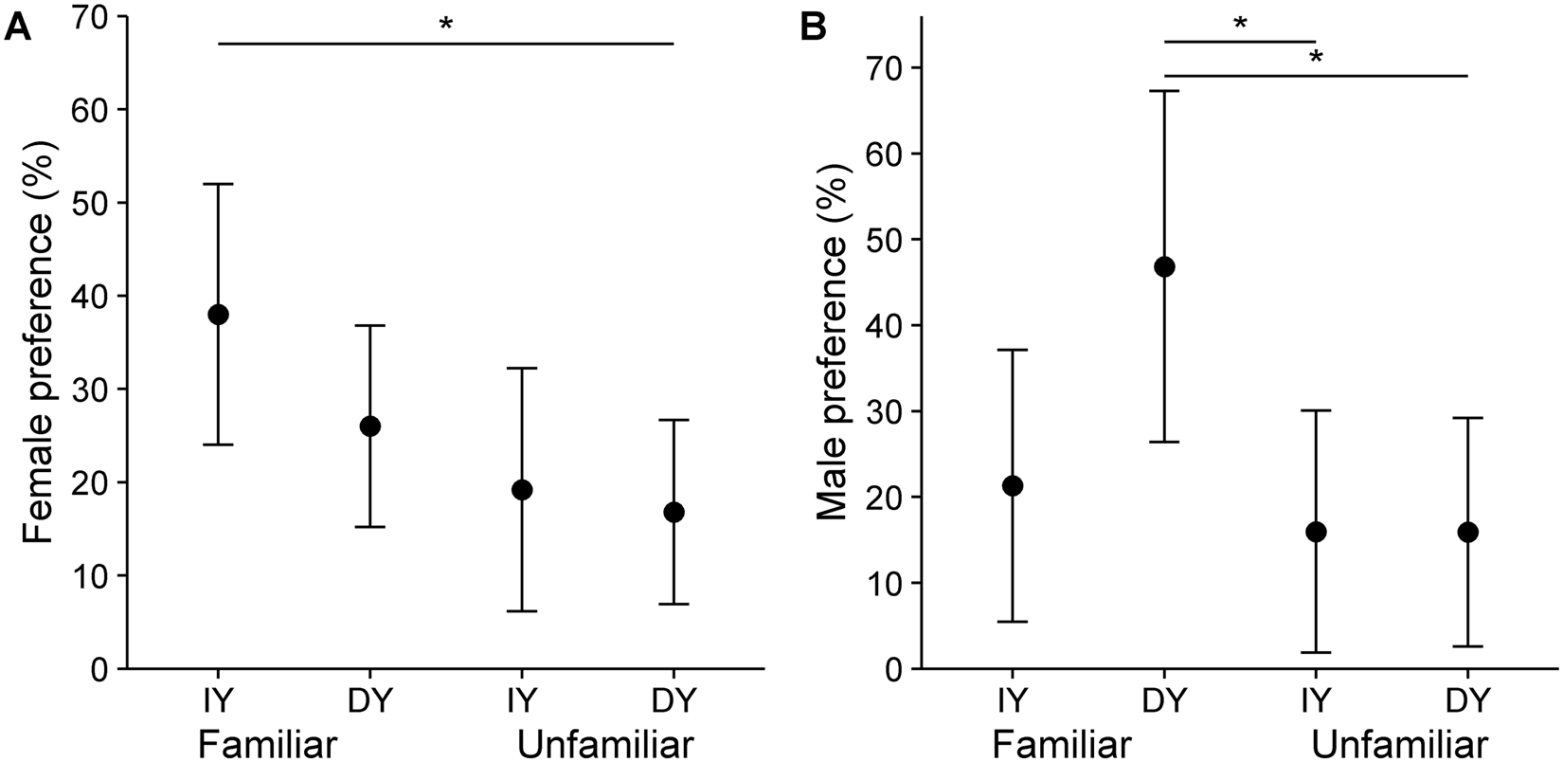
Results of social choice experiments on white-eyed bulbuls (*Pycnonotus xanthopygos*) using four stimuli with increased or decreased yellow ventral plumage colour (IY: Increased yellow, ID: Decreased yellow) and familiarity. Means presented as dots with 95% CL. Significant differences are marked with an asterisk (Tukey’s post hoc test). (**A**) female social choice (**B**) male social choice.

Males and females were equally motivated, with no significant differences between sexes in the time they spent in the choice area (Welch’s t-test: *t*_*28.37*_ = −0.01, *P* = 0.99). In the social choice test, females preferred to associate with familiar females (Fig. 2A, Table 1 and Table S2). Males also preferred to associate with familiar males but also significantly preferred to associate with less ornamented males (Fig. 2B, Table 1 and Table S2).

## Discussion

We found that white-eyed bulbuls discriminate between familiar and unfamiliar conspecifics as well as variations in plumage colour intensity. Our results suggest that ornamentation and familiarity may be important features in both social and sexual selection processes. However, these two features seem to play different roles in males and females. More specifically, for females, both features interactively affect their mating decisions: on one hand, experimentally increasing the intensity of male yellow patches increased females’ sexual interest; on the other hand, this preference turned out to be significantly amplified when the owner of the colour-increased patch was an unfamiliar male.

Toth and Griggio^43^ found that a sexually selected trait can be important in a social context as well. The carotenoid-based throat patch, well known in the mutual mate choice of rock sparrows (*Petronia petronia*)^9^, has also been identified as the best predictor of leadership in foraging groups outside the reproductive period. Individuals with larger yellow patches are followed by more group-members and therefore more likely to initiate foraging bouts, probably because following good quality individuals can be beneficial in terms of increased likelihood of finding food (see below). Thus, besides its function in mate choice, this trait serves in this species’ group coordination and social organization. Similarly, in our study, female bulbuls, in seeking the company of other females, preferred familiar and more-yellow individuals, although the role of ornamentation seemed to be less important. Interestingly, in both species, ornaments are carotenoid-based. Carotenoids cannot be synthesized *de novo* but must be obtained from the diet, so one potential benefit of following a well-ornamented individual during social foraging would be to increase the probability of finding novel or high quality food, particularly carotenoid-rich food^44, 45^. Thus, carotenoid-based ornaments are very likely costly, honest quality indicators of the bearer^46, 47^, and probably provide similar information about quality in both social and sexual contexts. This would predict that males would prefer social partners with brighter yellow patches, rather than duller yellow patches in social partners.

From the perspective of sexual selection, male bulbuls differ from females. For males, mate choice familiarity or intensity of patch colour did not matter at all (see Results). Thus, mutual mate choice does not seem to exist in bulbuls, at least with respect to the two features investigated. This is surprising given that female plumage ornaments are as prominent as those of males.

In the social context, the importance of one trait differed between the sexes. While the social choice tests revealed a preference for familiar individuals by both sexes, they behaved differently concerning the importance of the plumage ornament. Unlike females, males preferred to associate with less ornamented familiar males. From an evolutionary perspective, such a choice would make sense if familiar means also genetically more similar. Familiarity is also useful even if kinship is not involved, as it helps to reduce negative interactions, and might enhance foraging through observational learning. That is, joining familiar males may be beneficial from an inclusive fitness perspective. One possible explanation for the choice of less-ornamented social companions could be related to reducing aggressive interactions and improving dominance status. Indeed, in some species, for example the golden-crowned sparrows (*Zonotrichia atricapilla*)^48^ and rock sparrows^9^, individuals use carotenoid-based patches to signal fighting ability (“badge of status”). Whether the yellow patch is a badge of status in bulbuls remains unknown. Alternatively, seeking the company of less attractive social members may be ultimately linked to the sexual context in that joining less attractive competitors may increase a male’s own mating chances. Recently, Gasparini *et al*.^49^ have shown that male guppies (*Poecilia reticulata*) prefer females surrounded by unattractive males over females surrounded by attractive males.

Several hypotheses may explain individual preferences for unfamiliar mating partners. It may be a basic mechanism in species at high risk of inbreeding. In line with this, the specific dispersal patterns of group-living social species^50^, avoidance of kin as mates^51^, and mating with extra-group partners^52^ have each been identified as mechanisms to minimize inbreeding^53^. Thus, an ability to identify the degree of familiarity may be essential for kin avoidance when searching for mating partners (sexual context)^54^. Another hypothesis suggests that when mate choice is based on non-additive genetic traits, individuals can benefit by choosing genetically dissimilar mates (the compatible mate hypothesis) or more heterozygous mates (the heterozygous mate hypothesis)^55–57^. Furthermore, females may prefer unfamiliar mating partners to increase offspring heterozygosity. In monogamous black-legged kittiwakes (*Rissa tridactyla*), it was found that pairs were more genetically dissimilar than expected by chance^58^. Moreover, genetic similarity of pairs was negatively correlated with the number of chicks hatched while offspring heterozygosity was positively correlated with growth rate and survival. Thus, females may tend to increase their offspring’s heterozygosity by mating with unfamiliar mates (genetic similarity avoidance). Similarly, Marshall *et al*.^59^ found in the sedge warbler (*Acrocephalus schoenobaenus*) and Oh and Badyaev^60^ in the house finch (*Carpodacus mexicanus*) that females paired significantly more often with genetically dissimilar males.

In conclusion, our results indicate differential use of signal categories. That is, because honest signals like ornamentation convey the same information in both social and sexual contexts, they are used in a similar way. In contrast, traits signalling other characteristics like relatedness or familiarity may be differently perceived in sexual or social contexts. Furthermore, they may play different roles in determining male and female preferences in each context.

## Methods

### Subjects and housing

The experimental birds were captured in the surroundings of Antalya (south-west Turkey). In October 2013, 70 bulbuls were captured in Karatepe District (36°52′57.35″N, 30°32′54.59″E) located in western Antalya, and a further 24 bulbuls in November 2014 from Kurşunlu District (37°2′22.42″N, 30°48′4.36″E) located in northern Antalya about 28 km from the former location. The birds were individually marked with a unique combination of colour rings and aluminium ring. All birds were housed in outdoor aviaries in Antalya in groups of 6 to 7 birds of the same sex (aviary size: 8 m × 4 m × 3.0 m). All aviaries were equipped in the same way with vegetation, seven perches and two trees. Commercial food for granivorous passerines (Chicken food, Kardeşler Yem San ve Tic. A.Ş.), fresh fruit, mealworms and water were provided *ad libitum*. Birds captured in the second area were considered “unfamiliar” in respect to birds captured in the first area. Unfamiliar birds were kept visually and acoustically isolated in outdoor aviaries (36°56′50.59″N, 30°37′54.45″E) 500 m away from familiar birds (36°57′0.26″N, 30°38′10.99″N). At the time of the experiment all birds were more than a year old.

### Experimental design

Preference tests were conducted during April and May 2015 using an outdoor four-choice aviary (8 m × 4 m × 3.0 m, see Fig. S2). The four choice chambers hosting the stimuli were separated by opaque dividers i) to prevent visual interaction between them and ii) to prevent the focal individual from simultaneously observing two or more stimuli when in the choice area (see Fig. S2). The chamber of the stimuli and the choice area of the focal individuals were separated by a wire mesh to prevent close contact while allowing the focal individual to observe the stimulus. Observation was further facilitated by a perch, positioned in front of each of the four chambers.

At the beginning of each trial, the focal and stimulus individuals were placed in their experimental chambers and allowed at least 15 minutes to acclimatize before the trial began. The position of the focal bird was then recorded for 2 hours. Trials were broadcast via a video camera to a monitor in a nearby office to ensure that bird behaviour was not influenced by the presence of an observer. We measured the time a focal bird spent in the choice area in front of a particular stimulus compartment. Preference was expressed as the percentage of time in front of each stimulus relative to the total time in the choice area (choice time). We defined the preferred stimulus as the individual with whom the focal bird spent the most time.

In accordance with the objectives of the study, the focal individual had a choice between four stimuli: familiar with increased yellow, familiar with decreased yellow, unfamiliar with increased yellow and unfamiliar with decreased yellow. To control for a potential position effect, chambers were randomly assigned to stimulus individuals.

To distinguish between the effect of the yellow ventral patch and other attributes of individual attractiveness, the intensity of the yellow patch colour was manipulated with individuals randomly assigned to either an increased or decreased yellow group. The increased yellow (artificially enhanced) stimuli were treated with yellow and the decreased yellow (artificially dulled) stimuli with white non-toxic hair colours (Xuchang Shengyuan Hair Products Co., Ltd.) a couple of hours before the choice test. We changed the ventral yellow patch colour, by gently painting the plumage surface with the non-toxic hair colours, previously dampened. All the manipulations were performed in the natural range of ventral yellow patch colours (authors’ personal observations). Observations performed on birds after manipulation revealed no signs of stress. Although the choice area was only 5.34% of the experimental aviary area, the focal birds spend on average 56.99% of their time in the choice area.

In the two mate choice experiments (all stimuli were of the opposite sex to each focal bird), 18 focal females and 15 focal males were used. Stimuli were used several times but in each trial a different stimulus set of individuals was used, being randomly assigned to the two ornament manipulation treatments. For the mate choice experiments, 27 males (mean = 2.67, range = 1–6 times per individual) and 23 females (mean = 2.61, range = 1–6 times per individual) were used as the stimulus. In the two social choice experiments (all stimuli were of the same sex as the focal bird), 19 females and 12 males were used, with 21 males (mean = 2.29, range = 1–4 times per individual) and 24 females (mean = 3.17, range = 2–8 times per individual) being used as the stimulus. Focal birds were used only once per experiment so the number of focal birds per experiment indicates the number of trials.

### Molecular sexing, morphological measurements and spectrometry

To ensure correct sex determination of monochromatic white-eyed bulbuls, molecular sexing was performed using *P2/P8* primers^61, 62^. Standard measurements of wing length to the nearest 0.5 mm and tarsus length to the nearest 0.1 mm were taken. Body mass was recorded to the nearest 0.1 g were taken. Yellow ventral patch (see Fig. S1) of each male and female were measured before and after manipulation. We used an Ocean Optics S2000 Spectrometer (Eerbek, The Netherlands) with ultraviolet (deuterium) and visible (tungsten-halogen) lamps (Ocean Optics DH-2000-BAL, light source) and a bifurcated fiber optic probe. The fiber optic probe both provided illumination and obtained light reflected from the sample.

The probe was held at a 90° angle to the measured surface and ambient light was excluded using a black tube that held the probe tip at 4 mm distance from the surface. Five consecutive measurements were taken, lifting and replacing the probe each time, and then averaged for each individual^63^. Before measurement of each individual the spectrometer was recalibrated using a standard white (WS-2); for calibration of black the probe was removed from the light source and the cap of the plug closed. Spectral data were then processed in the ‘pavo’ package of R statistical program version 3.1.2^64, 65^. Calculations were carried out for reflectance in the 300–700 nm range. Relative reflectance in the UV (300–400 nm) and yellow (550–625 nm) parts of the spectrum were used in the analyses. UV-chroma was calculated as UV reflectance as a proportion of total reflectance (R300–400 nm /R300–700 nm). Yellow-chroma was calculated as the percentage of total light reflected in the range 550–625 nm^66^.

### Statistics

Outcomes from both preference experiments were analysed using analysis of variance (ANOVA), in which preference (percentage of time in front of each stimulus relative to the total time in the choice area) was the dependent variable, with two factors (familiarity and yellow) and their interaction included in the model. Arcsine transformation was applied to the preference to ensure that they met the assumptions of parametric statistics. Assumption of homogeneity of variance for all models tested by Levene’s test was satisfied after the transformation^67^. Normality of residuals of the model visually assessed by Q–Q plots improved after transformation but in some cases was not perfect. Omega-squared (ω^2^) was calculated as an estimate of effect size^67^. Statistical analyses were performed with R statistical program version 3.1.2^64^. All tests are two-tailed.

### Ethics

This study was conducted with the permission of the Ministry of Environment and Forestry (17825/2011) and according to Akdeniz University Ethical Committee on Animal Experiments regulations (134/2012).

## Electronic supplementary material

Supplemental information
