# Supplementary material for: Sexual and non-sexual social preferences in male and female white-eyed bulbuls
Source: Sci Rep. 2017 Jul 19;7:5847. doi: 10.1038/s41598-017-06239-3 (PMC5517633; doi:10.1038/s41598-017-06239-3)
Supplement: Supplementary file 1 — Supplemental information [file 41598_2017_6239_MOESM1_ESM.docx]

**Supplementary Information**

**Sexual and non-sexual social preferences in male and female white-eyed bulbuls**

Bekir Kabasakal, Miroslav Poláček, Aziz Aslan, Herbert Hoi, Ali Erdoğan, Matteo Griggio


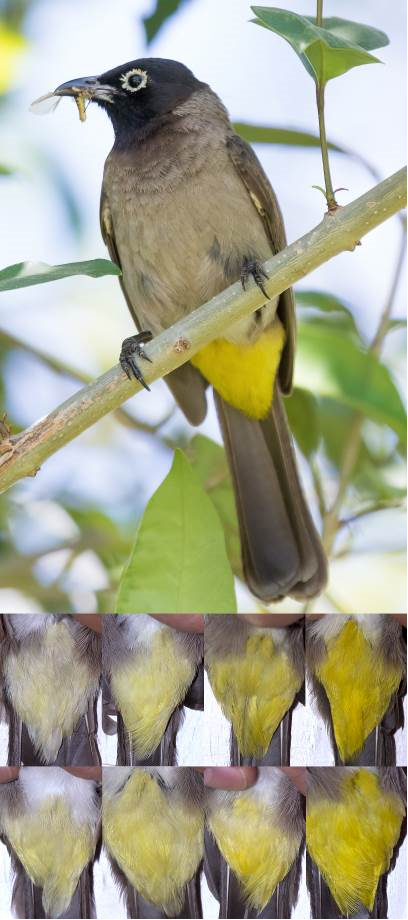


Figure S1. White-eyed bulbuls (*Pycnonotus xanthopygos*) and natural variation of yellow patch. Upper row are females and lower row males. Credit: Miroslav Poláček.


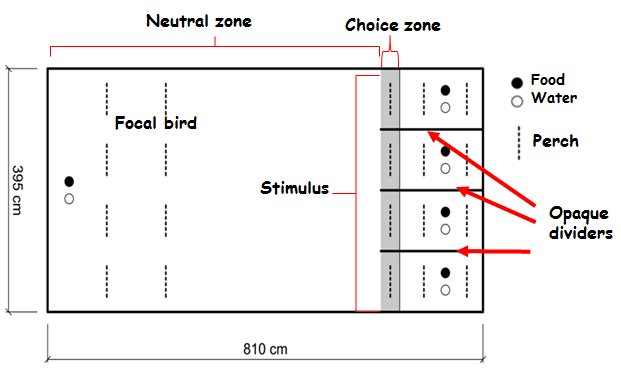


**Figure S2.** Schematic overview of the experimental aviary. Experimental aviary used for the sexual choice and social choice experiments on white-eyed bulbuls (*Pycnonotus xanthopygos*) using four stimuli (familiar or unfamiliar) with increased or decreased yellow ventral plumage colour.

**Table S1.** Results of ANOVA post hoc test (Tukey honestly significant difference test) comparing preference of experimental birds, white-eyed bulbuls (*Pycnonotus xanthopygos*), for familiarity and ornament colouration (increased, IY, or decreased, DY, yellow ventral plumage colour) in mate choice experiment, using four different stimuli (male mate choice results are not shown due to non-significant results from the ANOVA, see Results section).

| **Experiment** | **Stimulus category** | | | **Difference** | **Lower** | **Upper** | **P** |
| --- | --- | --- | --- | --- | --- | --- | --- |
| Female mate choice | Familiar IY | vs | Familiar DY | -0.008 | -0.224 | 0.208 | 1.000 |
|  |  |  | Unfamiliar IY | **0.328** | 0.112 | 0.544 | **0.001** |
|  |  |  | Unfamiliar DY | -0.054 | -0.27 | 0.162 | 0.912 |
|  | Familiar DY | vs | Unfamiliar IY | **0.336** | 0.12 | 0.551 | **0.001** |
|  |  |  | Unfamiliar DY | -0.046 | -0.262 | 0.17 | 0.942 |
|  | Unfamiliar IY | vs | Unfamiliar DY | **-0.382** | -0.598 | -0.166 | **0.000** |

**Table S2.** Results of ANOVA post hoc test (Tukey honestly significant difference test) comparing preference of experimental birds, white-eyed bulbuls (*Pycnonotus xanthopygos*), for familiarity and ornament colouration (increased, IY, or decreased, DY, yellow ventral plumage colour) in social choice experiment, using four different stimuli.

| **Experiment** | **Stimulus category** | | | **Difference** | **Lower** | **Upper** | **P** |
| --- | --- | --- | --- | --- | --- | --- | --- |
| Female social choice | Familiar IY | vs | Familiar DY | -0.138 | -0.414 | 0.137 | 0.552 |
|  |  |  | Unfamiliar IY | -0.275 | -0.55 | 0.001 | 0.051 |
|  |  |  | Unfamiliar DY | **-0.281** | -0.557 | -0.006 | **0.044** |
|  | Familiar DY | vs | Unfamiliar IY | -0.136 | -0.412 | 0.139 | 0.564 |
|  |  |  | Unfamiliar DY | -0.143 | -0.418 | 0.133 | 0.526 |
|  | Unfamiliar IY | vs | Unfamiliar DY | -0.006 | -0.282 | 0.269 | 1.000 |
| Male social choice | Familiar IY | vs | Familiar DY | 0.350 | -0.003 | 0.704 | 0.053 |
|  |  |  | Unfamiliar IY | -0.069 | -0.422 | 0.285 | 0.954 |
|  |  |  | Unfamiliar DY | -0.041 | -0.394 | 0.312 | 0.990 |
|  | Familiar DY | vs | Unfamiliar IY | **-0.419** | -0.772 | 0.065 | **0.014** |
|  |  |  | Unfamiliar DY | **-0.391** | -0.745 | -0.038 | **0.025** |
|  | Unfamiliar IY | vs | Unfamiliar DY | 0.028 | -0.326 | 0.381 | 0.997 |
